# An early bothremydid (Testudines, Pleurodira) from the Late Cretaceous (Cenomanian) of Utah, North America

**DOI:** 10.7717/peerj.2502

**Published:** 2016-09-28

**Authors:** Walter G. Joyce, Tyler R. Lyson, James I. Kirkland

**Affiliations:** 1 Departement für Geowissenschaften, Universität Freiburg, Freiburg, Switzerland; 2 Department of Earth Sciences, Denver Museum of Nature and Science, Denver, CO, USA; 3 Utah Geological Survey, Salt Lake City, UT, USA

**Keywords:** Late cretaceous, Paleobiogeography, Cenomanian, Utah, Naturita formation, Testudines, Pleurodira, Bothremydidae, *Paiutemys tibert*

## Abstract

**Background:**

Bothremydidae is a clade of extinct pleurodiran turtles known from the Cretaceous to Paleogene of Africa, Europe, India, Madagascar, and North and South America. The group is most diverse during the Late Cretaceous to Paleogene of Africa. Little is known, however, about the early evolution of the group.

**Methods:**

We here figure and describe a fossil turtle from early Late Cretaceous deposits exposed at MacFarlane Mine in Cedar Canyon, southwestern Utah, USA. The sediments associated with the new turtle are utilized to infer its stratigraphic provenience and the depositional settings in which it was deposited. The fossil is compared to previously described fossil pleurodires, integrated into a modified phylogenetic analysis of pelomedusoid turtles, and the biogeography of bothremydid turtles is reassessed. In light of the novel phylogenetic hypotheses, six previously established taxon names are converted to phylogenetically defined clade names to aid communication.

**Results:**

The new fossil turtle can be inferred with confidence to have originated from a brackish water facies within the late Cenomanian Culver Coal Zone of the Naturita Formation. The fossil can be distinguished from all other previously described pleurodires and is therefore designated as a new taxon, *Paiutemys tibert* gen. et. sp. nov. Phylogenetic analysis places the new taxon as sister to the European *Polysternon provinciale*, *Foxemys trabanti* and *Foxemys mechinorum* at the base of Bothremydinae. Biogeographic analysis suggests that bothremydids originated as continental turtles in Gondwana, but that bothremydines adapted to near-shore marine conditions and therefore should be seen as having a circum-Atlantic distribution.

## Introduction

Bothremydidae is an extinct clade of pelomedusoid turtles. The group was originally known from rare fossil material from the Late Cretaceous to Paleogene of North America (see Hay, 1908 for summary), but more recent efforts yielded a surprising diversity of fossil forms ranging from the Early Cretaceous to Paleogene of North and South America, Europe, Africa, India, and Madagascar, most recently summarized by Gaffney, Tong & Meylan (2006). In contrast to extant representatives of the clade Pelomedusoides, which only occupy freshwater aquatic habitats and possess limited diversity and morphological disparity (Ernst & Barbou, 1989), bothremydids are notable for exhibiting high levels of cranial disparity indicative of generalist, molluscivorous, and piscivorous diets and for inhabiting freshwater, estuarine, and costal environments (Gaffney, Tong & Meylan, 2006). The partial to full adaptation to salt water allowed some groups of bothremydids to disperse across various oceanic barriers that were developing during the Cretaceous (e.g., Rabi, Tong & Botfalvai, 2012; Pérez García, 2016).

In additional to reviewing the taxonomic and phylogenetic relationships of the group, Gaffney, Tong & Meylan (2006) provided a taxonomy that divided Bothremydidae into four primary clades: the Indian Kurmademydini, the South American and African Cearachelyini, and the circum-Atlantic Bothremydini and Taphrosphyini (Gaffney, Tong & Meylan, 2006; with notable additions from Cadena, Bloch & Jaramillo, 2012). Subsequent analyses have since concluded that the topology of Gaffney, Tong & Meylan (2006) is easily perturbed through minor modifications, especially through the addition of new taxa (e.g., Romano et al., 2014; Cadena, 2015). This, in return, makes the proposed nomenclatural system of Gaffney, Tong & Meylan (2006) difficult to apply. However, all phylogenetic hypotheses imply that the diversification of the group occurred in the early Late Cretaceous, despite the fact that the vast majority of finds are known from the late Late Cretaceous and Paleogene. This pattern also holds true for North America, as the earliest previously reported bothremydid, a poorly dated shell referable to *Chedighaii barberi*, (Schmidt, 1940) is at most Coniacian in age (Gaffney, Tong & Meylan, 2006).

The surprising discovery of a small-bodied bothremydid in Cenomanian deposits in Utah provides new insights into the phylogeny and biogeography of the group. The purpose of this contribution is to describe the fossil as a new species of bothremydid turtle and to investigate the phylogenetic relationships and biogeographic distribution of bothremydid turtles. We furthermore propose an internally consistent set of phylogenetically defined clade names to facilitate communication.

## Geological Setting

The new bothremydid fossil, Natural History Museum of Utah (UMNH) VP26151, was re-discovered by JIK and Don DeBlieux (Utah Geological Survey) in the collections of Zion National Park in 2003. The associated records indicate that the specimen had been collected outside of the park at MacFarlane Mine in the early 1960s, but the specimen had apparently lingered unprepared in the collections for decades. At its re-discovery, the turtle was embedded in a crumbling slab of dark-grey, fossiliferous, silty shale with identifiable gastropods and bivalves.

**Figure 1:**
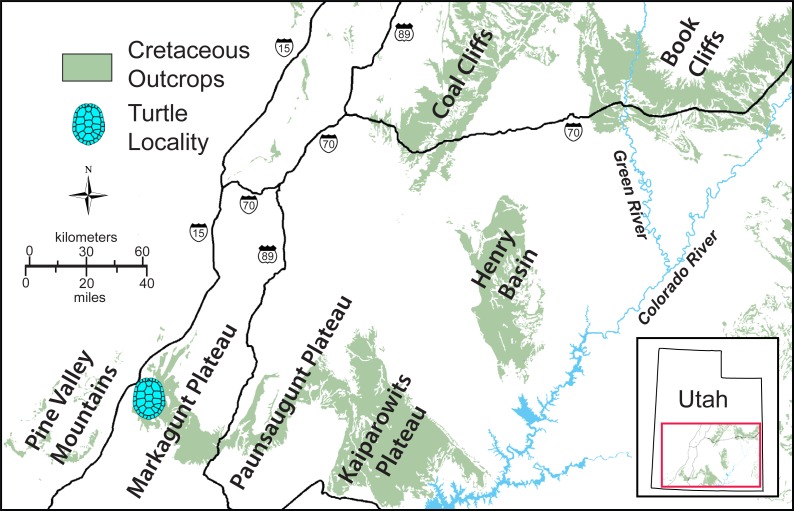
Simplified map of southwestern Utah. The exposure of Cretaceous sediments and the approximate placement of the type locality of *Paiutemys tibert* gen. et sp. nov. near the MacFarlane Mine in Cedar Canyon are highlighted.

**Figure 2:**
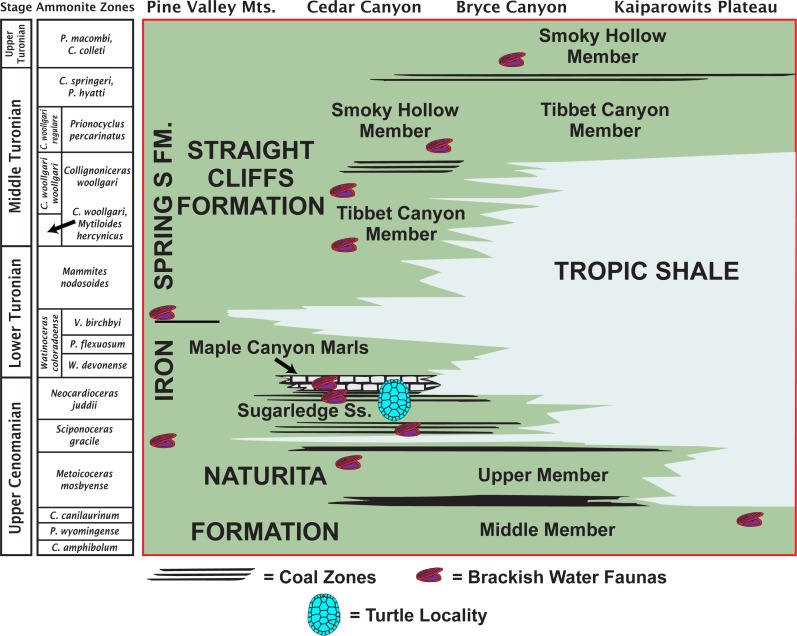
Late Cretaceous stratigraphy of southwestern Utah. Stratigraphic summary of early Late Cretaceous (Cenomanian–Turonian) sediments exposed in southwestern Utah highlighting the likely stratigraphic placement of the type locality of *Paiutemys tibert* gen. et sp. nov. (modified from Tibert et al., 2003).

The MacFarlane Mine (UMNH VP.LOC.2141) is located in Cedar Canyon, just south of Utah State Highway 14, in the western regions of the Markagunt Plateau of southwestern Utah (Doelling, 1972; Fig. 1). A massive landslide covered the area in recent years and the mine is no longer accessible. Fortunately, the mid-Cretaceous stratigraphy had previously been studied in considerable detail (Eaton et al., 2001; Tibert et al., 2003; Laurin & Sageman, 2007) and it is therefore possible to date the fossil with confidence based on the associated lithology and fossil invertebrates. The accompanying molluscs include juvenile or dwarfed specimens of the gastropods *Admetopsis* n. sp. B (Eaton et al., 2001; Hoffman, 2005) and *Cassiope* (= *Craginia*) *utahensis* (Mennessier, 1984; Eaton et al., 2001) and of the bivalve *Carycorbula nematophora* (Kirkland, 1996). The small size (<0.5 cm), low diversity, and subdued ornamentation of this molluscan assemblage suggest a mesohaline to lower brachyhaline salinity on the order of 0.5–2.0 percent (Fürsich & Kirkland, 1986; Fürsich, 1994). The turtle is therefore confidently interpreted as originating from a brackish water facies at the top of the Culver Coal Zone of the Naturita Formation (formerly Dakota Formation; Young, 1960; Carpenter, 2014) in the Maple Canyon Marl Zone (Tibert et al., 2003), which corresponds to the uppermost Cenomanian (Late Cretaceous) *Neocardioceras juddii* ammonite biozone (Tibert et al., 2003; Fig. 2).

## Phylogenetic Nomenclature

### *Bothremydidae* Baur, 1891, converted clade name

Definition. *Bothremydidae* refers to the most inclusive clade containing *Bothremys cooki* Leidy, 1865, but not *Pelomedusa subrufa* (Bonnaterre, 1789) or *Podocnemis expansa* (Schweigger, 1812).

### *Bothremydinae* Williams, 1950, converted clade name

Definition. *Bothremydidae* refers to the most inclusive clade containing *Bothremys cooki*, but not *Pelomedusa subrufa*, *Podocnemis expansa*, or *Kurmademys kallamedensis* Gaffney, Chatterjee & Rudra, 2001.

### *Bothremydini* Gaffney, Tong & Meylan, 2006, converted clade name

Definition. *Bothremydini* refers to the most inclusive clade containing *Bothremys cooki*, but not *Cearachelys placidoi* Gaffney, Almeida Campos & Hirayama, 2001, *Kurmademys kallamedensis*, or *Taphrosphys sulcatus* (Leidy, 1856).

### *Cearachelyini* Gaffney, Tong & Meylan, 2006, converted clade name

Definition. *Cearachelyini* refers to the most inclusive clade containing *Cearachelys placidoi*, but not *Bothremys cooki*, *Kurmademys kallamedensis*, or *Taphrosphys sulcatus*.

### *Kurmademydini* Gaffney, Tong & Meylan, 2006, converted clade name

Definition. *Kurmademydini* refers to the most inclusive clade containing *Kurmademys kallamedensis*, but not *Bothremys cooki*, *Cearachelys placidoi*, or *Taphrosphys sulcatus*.

### *Taphrosphyini* Gaffney, Tong & Meylan, 2006, converted clade name

Definition. *Taphrosphyini* refers to the most inclusive clade containing *Taphrosphys sulcatus*, but not *Bothremys cooki*, *Cearachelys placidoi*, or *Kurmademys kallamedensis*.

### Comments

The name Bothremydidae was originally coined by Baur (1891) for a poorly circumscribed set of fossil turtles from North America, but was only used irregularly over the course of the following decades, likely because a lack of skull material prohibited a rigorous classification of these taxa (e.g., Schmidt, 1940). Williams (1950) was the first to apply the name to a subfamily and we therefore assign authorship to him for that exact spelling according to the rationale outlined by Joyce, Parham & Gauthier (2004). Following renewed interest in the group, the name was resurrected, first as a subfamily (Gaffney, 1975) and then as a family (Antunes & Broin, 1988). The latter usage has remained relatively stable ever since, though an ever-increasing number of new taxa have led to a significant expansion of the group. The phylogenetic analysis of Gaffney, Tong & Meylan (2006) demanded a revised nomenclatural system. Although many solutions were available within the confines of the International Commission of Zoological Nomenclature (1999), such as the creation of numerous families, we find the solution of naming four subclades (tribes) as done by Gaffney, Tong & Meylan (2006) more reasonable, as this maintained existing nomenclatural practice. However, given that the International Commission of Zoological Nomenclature (1999) does not allow fixing the content of a taxon, the current system is prone to instability, either through the justified need to accommodate changes to the tree or through the unjustified need to create new taxon names for their own sake.

We are confident that the vast majority of paleontologists would maintain the current names if a single taxon or subclade were to switch its position and as long as these do not include the type of another clade. Indeed, Romano et al. (2014) and Cadena (2015) already applied these names in this fashion. We therefore fix the usage of Gaffney, Tong & Meylan (2006) by tying the six primary names, Bothremydidae, Bothremydinae, Bothremydini, Cearachelyini, Kurmademydini, and Taphrosphyini to six stem clades that are defined relative to each other. We note that it is not necessary or desirable to name every clade as it hinders efficient communication, so we bypass fixing the meaning of such silly nomenclatural constructs as Bothremydinura or Bothremydodda (Gaffney, Tong & Meylan, 2006).

## Nomenclatural Acts

The electronic version of this article in Portable Document Format (PDF) will represent a published work according to the ICZN, and hence the new names contained in the electronic version are effectively published under that Code from the electronic edition alone. This published work and the nomenclatural acts it contains have been registered in ZooBank, the online registration system for the ICZN. The ZooBank LSIDs (Life Science Identifiers) can be resolved and the associated information viewed through any standard web browser by appending the LSID to the prefix http://zoobank.org/. The LSID for this publication is: urn:lsid:zoobank.org:pub:8322CE62-F815-49A9-AC13-4169E4CD3F43. The online version of this work is archived and available from the following digital repositories: PeerJ, PubMed Central, and CLOCKSS.

## Systematic Paleontology

**Table:** 

PLEURODIRA Cope, 1865
PELOMEDUSOIDES Broin, 1988
BOTHREMYDIDAE Baur, 1891
BOTHREMYDINAE Williams, 1950

### *Paiutemys tibert* gen. et sp. nov.

**Figure 3:**
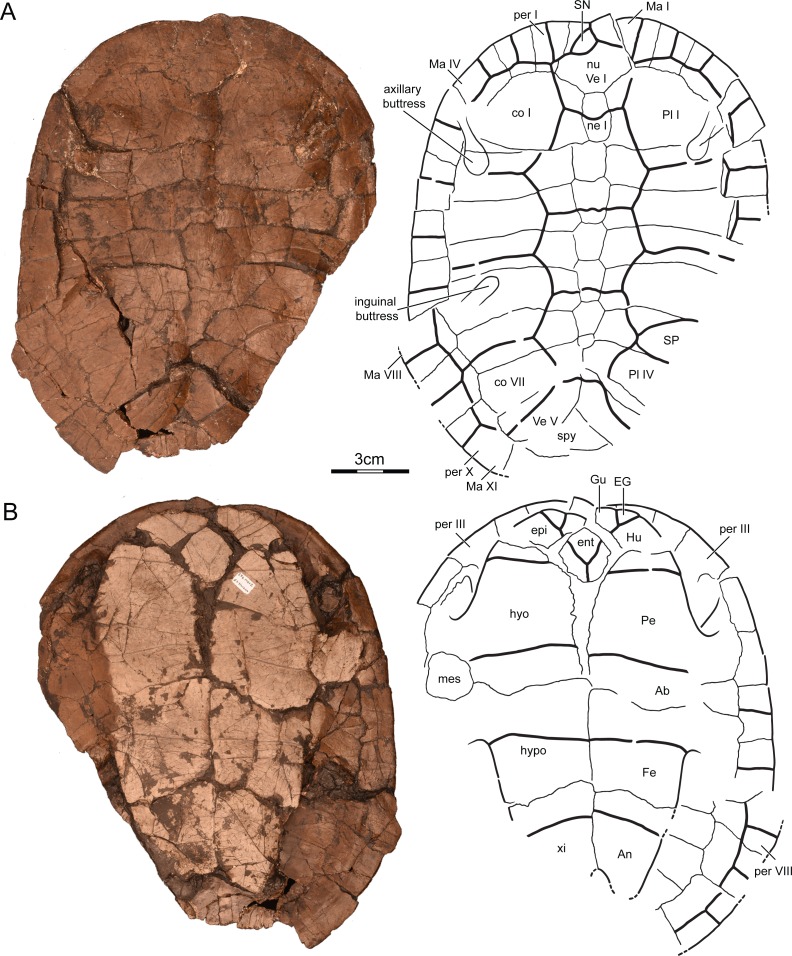
UMNH VP26151, *Paiutemys tibert* gen. et sp. nov., holotype, Late Cretaceous (Cenomanian) of Utah, USA. Photographs and illustrations of shell in (A) dorsal and (B) ventral view. Abbreviations: *Ab*, abdominal scute; *An*, anal scute; *co*, costal; *EG*, extragular scute; *ent*, entoplastron; *epi*, epiplastron; *Fe*, femoral scute; *Gu*, gular scute; *Hu*, humeral scute; *hyo*, hyoplastron; *hypo*, hypoplastron; *Ma*, marginal scute; *mes*, mesoplastron; *ne*, neural; *nu*, nuchal; *Pe*, pectoral scute; *per*, peripheral; *Pl*, pleural scute; *SP*, supernumerary pleural scute; *SN*, supernumerary nuchal scute; *spy*, suprapygal; *Ve*, vertebral scute; *xi*, xiphiplastron.

Holotype. UMNH VP26151, a nearly complete shell lacking right peripherals VII–XI, the distal portions of right costals IV–VIII, left peripheral XI, the pygal, and the margins of the right xiphiplastron (Fig. 3).

Type locality and horizon. MacFarlane Mine, south of Utah State Highway 14, east of Cedar City, Iron County, Utah (Fig. 1); top of the Culver Coal Zone, *Neocardioceras juddii* ammonite biozone, uppermost Cenomanian, Late Cretaceous (Fig. 2).

Etymology. The genus name is formed in allusion to the Southern Paiute (pronounced pie-yute), a group of indigenous people that natively inhabit parts of the southwestern United States, including the area surrounding Cedar City, Utah. The species name is formed in recognition of the late Neil E. Tibert, who was instrumental in establishing the stratigraphy of mid-Cretaceous beds in the type locality of the new taxon (Tibert et al., 2003). Following the rules of the International Commission of Zoological Nomenclature (1999), the species name is formed as a name in apposition and therefore not placed in the genitive.

Diagnosis. *Paiutemys tibert* gen et sp. nov. can be diagnosed as a pleurodire by the presence of a single gular scute, a deep anal notch, the lack of inframarginal scutes, and the likely sutural attachment of the pelvis to the shell. *Paiutemys tibert* can be diagnosed as a representative of *Bothremydidae* by the presence of a rectangular neural I, contact of the axillary buttress with the main body of peripheral III and of the inguinal buttress with costal V, reduced equidimensional mesoplastra, and by the absence of a cervical scale, and as a representative of *Bothremydinae* by the enlargement of costal I, reduction of the neural series to seven, and a midline contact of costals VII and VIII. *Paiutemys tibert* is distinguished from other representatives of *Bothremydinae* by the following combination of characters: presence of a nuchal notch (otherwise present in *Chedighaii barberi*, *Foxemys trabanti* Rabi, Tong & Botfalvai, 2012, “*Podocnemis*” *parva* Haas, 1978a, *Polysternon provinciale* (Matheron, 1869), and *Rosasia soutoi* Carrington da Costa, 1940), lack of an overlap of the pectoral onto the entoplastron (also present in *Rosasia soutoi*), lack of a pectoral overlap onto the epiplastra (broadly present among many other bothremydids), reduction of the anterior plastral lobe (otherwise present in “*Podocnemis*” *parva*, *Foxemys* spp., *Elochelys* spp., and *Polysternon provinciale*), and the apomorphic presence of a contact of the axillary buttress with costal II and of a surface sculpture consisting of a combination of fine striations in the central parts of the shell and fine polygons along the margins.

## Description

Preservation. UMNH VP26151is a relatively complete shell lacking right peripherals VII–XI, the distal portions of right costals IV–VIII, left peripheral XI, the pygal, and much of the margins of the right xiphiplastron (Fig. 3). The specimen was embedded in a coaly, clay-rich matrix and was strongly compacted during diagensis. Although some shell bones disarticulated and shifted during this process, it appears that the specimen mostly deformed plastically in the dorsoventral axis and therefore shows little distortion when viewed from dorsal or ventral. Four raised areas apparent in dorsal view are evidence of thickened portions of the shell that resisted plastic deformation. These include the outlines of the axillary buttresses on costals I and II, the outline of the left inguinal buttress on costal V, and the outlines of the pelvis on costals VIII. The vast majority of sulci are clearly discernable, with the exception of those presumably present along the bridge.

**Figure 4:**
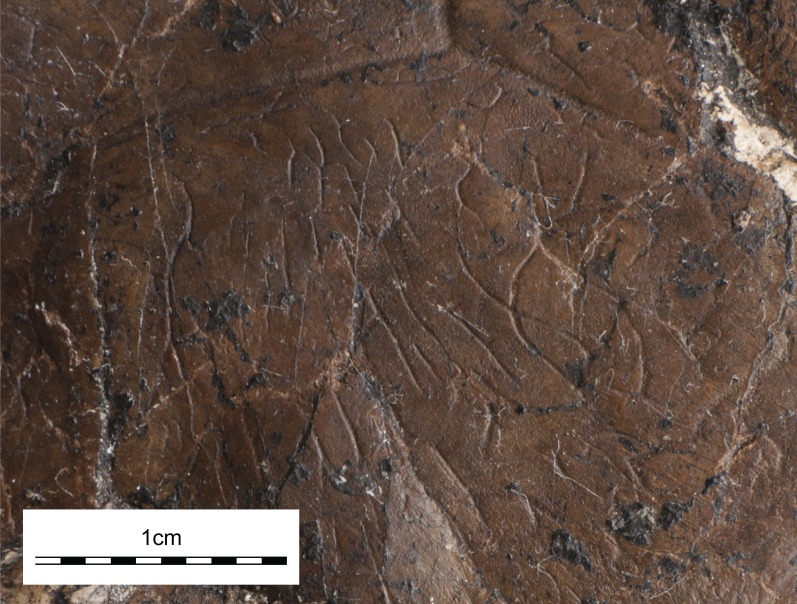
UMNH VP26151, *Paiutemys tibert* gen. et sp. nov., holotype, Late Cretaceous (Cenomanian) of Utah, USA. Close-up photograph of carapace at the intersection of vertebral I and II with right pleural I highlighting the shell texture in this region. The anterior of the specimen is oriented to the top.

Carapace. The preserved midline length of the shell is about 16.5 cm and we estimate that the carapace was originally about 18 cm long as only the pygal is missing (Fig. 3A). In dorsal view the shell is ovoid, but the anterior margin exhibits a shallow nuchal notch that spans between peripherals I. It is not possible to objectively establish the location of the greatest width of the shell, because the left peripheral series was distorted by compression. There is no sign of carinae or a lateral gutter. The surface of the shell is generally smooth, but the central portion of the carapace and plastron is decorated by fine, vermiculating striations that become denser towards the margins of the shell to form irregular polygons (Fig. 4).

Nuchal. The nuchal is a hexagonal element that is only slightly wider at its greatest width than its length along the midline, but it can nevertheless be classified as being roughly equidimensional (Fig. 3A). The anterior margin contributes to the broad but shallow nuchal notch and therefore traces the shape of a wide letter V. The nuchal has a broad anterolateral contact with peripheral I, a similarly broad posterolateral contact with costal I, and a short posterior contact with neural I.

Neurals. The neural series consists of seven contiguous elements. Neural I is an elongate rectangle, neurals II–VI are hexagonal with short anterior sides, whereas neural VII is irregular and button shaped (Fig. 3A). The neurals generally decrease in width and length from anterior to posterior, with exception of neural I, which is significantly narrower that neural II. Neural I only contacts a single pair of costals laterally, whereas neurals II–VI contact two pairs each. Neural VII is asymmetric and clearly contacts two costals on the right, but only the corner of a second costal on the left side.

Costals. The shell includes eight pairs of costals. The neural series does not permit any midline contact among the anterior elements, but reduction among the posterior neurals allows a midline contact of the posterior half of costals VII and the full length of costals VIII (Fig. 3A). Costals I are greatly enlarged elements that have an anteroposterior length that is more than twice that of costals II. As in the vast majority of other turtles, the remaining costals have similar anteroposterior lengths, become increasingly narrow towards the posterior, and increasingly rotate towards the posterior. The medial contacts of the costals with the neurals are listed above. Costals I laterally contact peripherals I–IV, costals V peripherals VII and VIII, costals VI peripherals VIII and IX, costals VII peripherals IX and X and costal VIII peripheral X and XI, but the lateral contacts of costals II–IV are obscured by crushing.

Peripherals. Although only ten peripherals are preserved, the size of the gap at the end of the peripheral ring is consistent with the former presence of an eleventh pair of peripherals in addition to a pygal (Fig. 3A). Peripherals I are wider anteriorly than posteriorly, but a slight asymmetry in the specimen renders the posterior side of the right peripheral shorter than that of the left peripheral. The bridge spans from the posterior third of peripherals III to the anterior third of peripherals VIII. Peripherals IV–VII were therefore likely V-shaped in cross section prior to crushing, whereas peripherals III and VIII act as intermediate elements.

Pygals and Suprapygals. Only a single, poorly preserved suprapygal is preserved along the posterior margin of the specimen (Fig. 3A). As in the vast majority of other pleurodires, it appears to have been a triangular element that lacked a lateral contact with peripheral X. The pygal is not preserved.

Carapacial Scutes. The carapace is inferred to have been covered by five vertebrals, four pairs of pleurals, twelve pairs of marginals and an irregular, supernumerary nuchal scute and an irregular, supernumerary pleural scute (Fig. 3A). A cervical is clearly absent. Vertebral I has an irregular shape due to the presence of the supernumerary scute, but it was likely pentagonal in regular specimens of the same taxon, as inferred from the regular, right side of the specimen. Vertebral I broadly contacts marginal I and the supernumerary scute anterolaterally, has a short lateral contact with marginal II, a broad posterolateral contact with pleural I, and a broad posterior contact with vertebral II. Vertebrals II–VI are hexagonal. Although the four posterior vertebrals are broader than vertebral I, there is a clear reduction in width towards the posterior. Each vertebral contacts two pleurals laterally, at least on the regular, left side of the specimen. The vertebral I/II sulcus runs over neural I and has a broad, posteriorly-oriented inflection. The vertebral II/III sulcus and the vertebral III/IV sulcus cross neurals III and V, respectively, and are ornamented with minor inflections. Vertebral V is about as wide as vertebral II. Given that the posterior contacts are not preserved, this element can only be described as posteriorly flaring.

Four pleural scutes are preserved on the left side of the specimen, but a fifth, irregular pleural is inserted between pleural III and IV on the right side of the specimen (Fig. 3A). The four regular pleurals on the left side of the specimen are generally broader than the vertebrals, each contact two vertebrals medially, and their interpleural sulci cross costals II, IV, and VI. Pleural I distally contacts marginals II–V, pleural II marginals V–VII, pleural III marginals VII–IX, and pleural IV marginals IX–XI. Marginals I and XII lack contacts with the pleurals.

Plastron. The plastron broadly covers the ventral aspects of the carapace, but does not protrude anteriorly beyond the margins of the carapace (Fig. 3B). The anterior plastral lobe is relatively short and broad, about twice as broad as long. The posterior plastral lobe, by contrast, is narrower and more elongate. The posterior marginals of the posterior lobe are damaged, but the anal notch can nevertheless be inferred to have been relatively deep.

Plastral Bones. The plastron consists of a pair of epi-, hyo-, meso-, hypo-, and xiphiplastra and a single entoplastron (Fig. 3B). The epiplastra are block shaped, have a short midline contact with one another and otherwise contact the entoplastron posteromedially and the hyoplastron posteriorly. The entoplastron is diamond shaped and contacts the epiplastral anterolaterally and the hyoplastral posterolaterally. The hyo-, hypo-, and xiphiplastra combined form the vast majority of the plastron. The axillary buttress can clearly be seen to have contacted the posterior third of peripheral III, but crushing obscures the visceral aspects. However, symmetrically raised areas on the dorsal side of the specimen allow inferring a contact of the buttress with costals I and II. The inguinal buttress can similarly be observed to contact the anterior third of peripheral VIII in ventral view, but it appears to have broadly contacted costal V as inferred by raised areas apparent in dorsal view. The mesoplastra are reduced in size, approximately as long as wide, and laterally contact peripherals V and VI.

Plastral Scutes. We here follow the nomenclatural system of Hutchison & Bramble (1981) by naming the gular scutes ’gulars’ and ’extragulars.’ A single gular and a pair of extragulars, humerals, pectorals, abdominals, femorals, and anals cover the plastron (Fig. 3B). The gular is a relatively large element that covers the anterior two-thirds of the entoplastron and the lateral third of the epiplastra. The extragulars are small elements that are restricted to the epiplastra and do not contact one another along the midline. The humerals have a short medial contact with one another and block the pectorals from covering the entoplastron. The midline contacts of the pectorals, abdominals, femorals are similar in size, but that of the anals is much shorter. Sulci are poorly preserved in the bridge region and it is therefore difficult to discern the lateral contacts of the plastral scutes, but inframarginals nevertheless appear to be missing.

Pelvis. The shell is flattened and it is therefore not possible to observe the vast majority of internal structures (Fig. 3B). However, as described above, raised areas allow inferring the presence and distribution of the axillary and inguinal buttresses. Unlike the plastral buttresses discussed above, these raised areas do not trace the underlying structures and were therefore omit them from the illustration (Fig. 3A). In the vast majority of turtles the pelvis is not suturally attached to the shell and therefore disarticulates quickly after death. We therefore furthermore interpret the raised areas below costals VIII as evidence of the suturally articulated pelvis typical of pleurodires.

## Phylogenetic Analysis

We explore the phylogenetic position of *Paiutemys tibert* gen. et sp. nov. by integrating the new turtle into the phylogenetic analysis of Gaffney, Tong & Meylan (2006), which was developed to investigate the relationships of pan-pelomedusoid turtles, particularly bothremydids. We performed a number of modifications to this matrix to allow ordering characters that form morphoclines. In particular, the sequence of character states was modified for characters 14 (temporal emargination), 28 (septum orbitotemporale), 32 (midline dorsal process), 40 (orbital-narial bar width), 60 (condylus mandibularis position), and 104 (basisphenoid-quadrate contact) to form morphoclines that then can be ordered. Character 139 (nuchal bone width) was rendered orderable by omitting character state 4, which scores the apomorphic condition apparent in *Araripemys barretoi* Price, 1973 that cannot be included in a morphocline. Instead this taxon was scored inapplicable for this character. Characters 38 and 39 of Gaffney, Tong & Meylan (2006; maxilla-quadratojugal contact and maxilla quadrate contact) were rendered orderable by splitting it into its component parts: presence of a maxilla-quadrate contact (character 38) and development of the cheek emargination (character 39). The two aspects that form character 51 of Gaffney, Tong & Meylan (2006; antrum postoticum), i.e., presence of an antrum postoticum and closure of the incisura columella auris, were similarly split into two separate characters. The former aspect forms an orderable character, whereas the latter aspect is redundant with character 52 (incisura columella auris) and was therefore omitted. Character 145 of Gaffney, Tong & Meylan (2006) (position of four-sided neural) was also rendered partially orderable, by splitting it into its components: the presence of a square neural (character 144) versus the placement of the square neural (character 145), which can be ordered. Character 149 (costal contact of axillary process) was already orderable, but was expanded to include the novel character state (axillary process in contact with costal 2) present in *Paiutemys tibert* gen. et sp. nov. Given that character 85 of Gaffney, Tong & Meylan (2006; exoccipital-quadrate contact) conflates two different characters (i.e., the presence and size of a exoccipital-quadrate contact and the ventral exposure of the prootic on the surface of the skull), we split this character into two separate characters as well, of which the first can be ordered, whereas the second is redundant with character 94 (ventral exposure of prootic). Character 148 (peripheral I-costal I contact) was finally rendered orderable as well by rearranging the character states and by omitting the autapomorphic condition for *A. barretoi*, which was scored inapplicable for this character. With exception of scoring *A. barretoi* non-applicable for three characters, and omitting redundant character components, these significant rearrangements to the matrix of Gaffney, Tong & Meylan (2006) are purely cosmetic, as they make no changes to the character observations coded by Gaffney, Tong & Meylan (2006). Instead, these modifications simply render 29 multistate characters orderable (i.e., 14, 20, 28, 32, 34, 38, 39, 40, 45, 47, 51, 56, 60, 68, 82, 85, 94, 104, 139, 140, 141, 145, 147, 148, 149, 150, 152, 159, and 171). That these modifications are cosmetic is easily demonstrated by the fact that the modified matrix retrieves the same results as Gaffney, Tong & Meylan (2006) when characters are left unordered. *Paiutemys tibert* could be scored for 32 of 176 characters. Throughout this paper, our characters are numbered starting with 1, not with 0, which is the default for TNT (Goloboff, Farris & Nixon, 2008).

For the first analysis, we followed the protocols of Gaffney, Tong & Meylan (2006) by excluding all shell taxa from the analysis. This preliminary analysis retrieves *Paiutemys tibert* near the base of Bothremydidae in the vicinity of the European bothremydids *Foxemys mechinorum* Tong, Gaffney & Buffetaut, 1998 and *Polysternon provinciale*. We therefore expanded the matrix for our second analysis to include additional taxa relevant to this part of the tree and to North American biogeography, in particular *Elochelys perfecta* Nopcsa, 1931 from the Campanian of France, *E. convenarum* Laurent, Tong & Claude, 2002 from the Maastrichtian of France, which had been shown by Gaffney, Tong & Meylan (2006) to be situated in this part of the tree, and by adding *Atolchelys lepida* from the Barremian of Brazil (as scored by Romano et al., 2014), *Chupacabrachelys complexus* from the Campanian of the Texas (as described by Lehman & Wick, 2010), and *Foxemys trabanti* from the Santonian of Hungary (as scored by Rabi, Tong & Botfalvai, 2012, including proposed modifications to the scoring of *F. mechinorum*). Given that the Triassic taxon *Proterochersis robusta* Fraas, 1913 was scored using faulty assumptions (Joyce, Schoch & Lyson, 2013), this basal turtle was omitted from the analysis. The final character list is provided in File S1 and the final character/taxon matrix for 47 active and 14 inactive taxa is provided in File S2.

**Figure 5:**
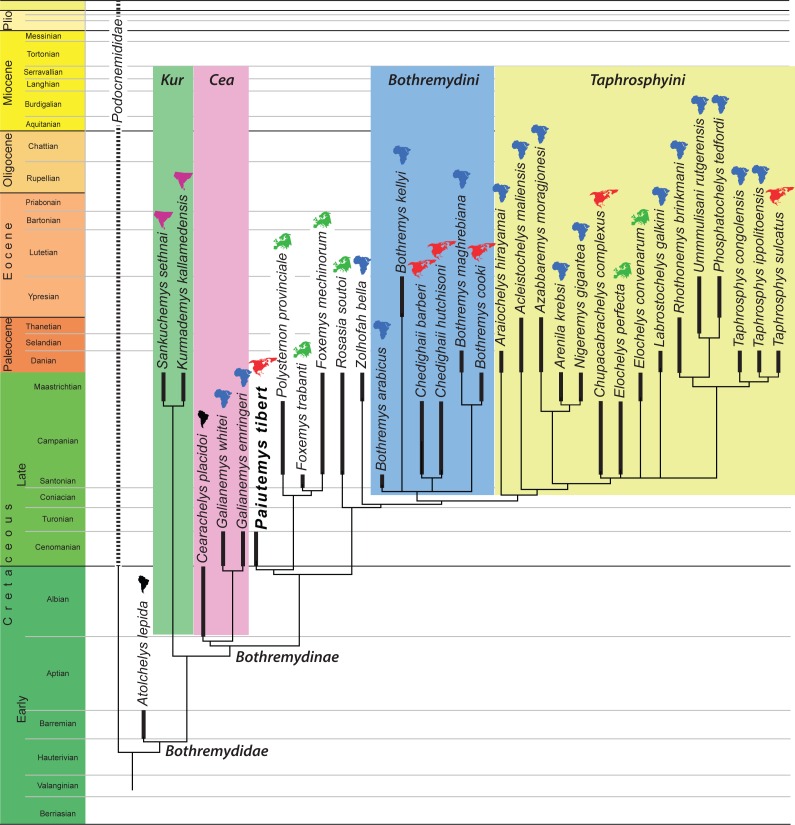
Phylogenetic hypothesis using ordered characters. A time-calibrated cladogram depicting the strict consensus topology of 30 equally parsimonious trees with 424 steps retrieved from the phylogenetic analysis using ordered characters. Dark lines highlight the known temporal distribution of a species and the colored continent symbols highlight the known spatial distribution. Abbreviations: *Cea*, Cearachelyini; *Kur*, Kurmademydini.

**Figure 6:**
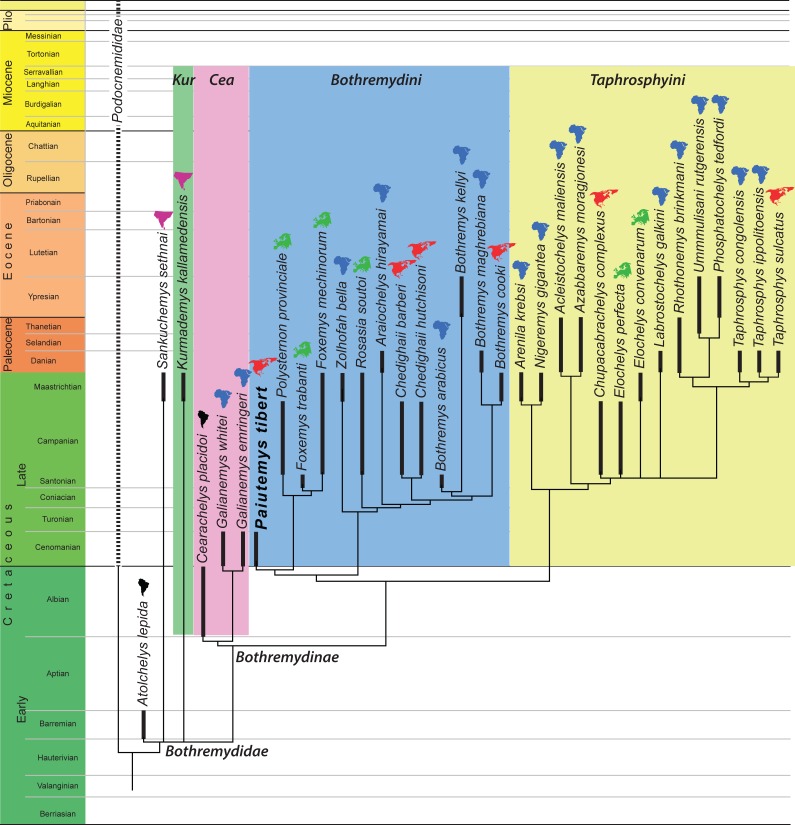
Phylogenetic hypothesis using unordered characters. A time-calibrated cladogram depicting the strict consensus topology of 27 equally parsimonious trees with 399 steps retrieved from the phylogenetic analysis using unordered characters. Dark lines highlight the known temporal distribution of a species and the colored continent symbols highlight the known spatial distribution. Abbreviations: *Cea*, Cearachelyini; *Kur*, Kurmademydini.

The final matrix was subjected to parsimony analysis using the software program TNT (Goloboff, Farris & Nixon, 2008) using 1,000 replicates of random addition sequences followed by a second round of tree bisection-reconnection. Two sets of analyses were performed. In the first analysis, all multi-state characters that form morphoclines (see above) were ordered. This analysis retrieved 30 most parsimonious trees with 424 steps. In the second analysis all characters were left unordered, which resulted in 27 most parsimonious trees with 399 steps. The strict consensus tree of the first analysis is provided in Fig. 5 and that of the second analysis in Fig. 6. The same trees with common synapomorphies are provided in Files S3 and S4.

## Discussion

### Alpha taxonomy

Our phylogenetic analyses (Fig. 5) place UMNH VP26151within the internested clades Pleurodira, Pelomedusoides, Bothremydidae, and Bothremydinae based on a substantial list of characters. These characters include the sutural attachment of the pelvis to the shell, a rectangular neural I, reduction of the neural series to seven elements combined with a midline contact of costals VII and VIII, enlargement of costal I to double the anteroposterior length of costal II, contact of the axillary buttress with the main body of peripheral III, contact of the inguinal buttress with costal V, absence of a cervical, a deep anal notch, a single gular scute, lack of inframarginal scutes, and reduced, equidimensional mesoplastra (Gaffney, Tong & Meylan, 2006).

Given that UMNH VP26151is one of only a few bothremydines recovered from the mid Cretaceous sediments world wide, we here differentiate UMNH VP26151relative to all described pre-Santonian bothremydines.

UMNH VP26151can be differentiated from the Albian *Cearachelys placidoi* by exhibiting a broader nuchal, absence of neural VIII, a square neural I, and a narrower vertebral I that only barely laps onto peripheral I (Gaffney, Tong & Meylan, 2006). Two shells have been reported from the Cenomanian of Morocco, American Museum of Natural History (AMNH) 35500 and AMNH 35501, that may reasonably belong to the two known species of *Galianemys* found in the same sediments (Gaffney, Tong & Meylan, 2006). UMNH VP26151differs from AMNH 35000 by having more hexagonal vertebrals with straight margins, a square neural I, the presence of neural VII, and a broader, more rounded anterior plastral lobe. UMNH VP26151similarly differs from AMNH 35001 having more pronounced hexagonal vertebrals, a square neural I, the absence of neural VIII, and a much larger gular scale. Inclusion of AMNH 35000 and AMNH 35001 in our matrix confirms their placement within Cearachelyini.

Two shells that serve as the holotypes of “*Podocnemis*” *parva* Haas 1978a and “*Podocnemis*” *judea* Haas 1978b have been reported from the Cenomanian of the West Bank. Gaffney, Tong & Meylan (2006) tentatively synonymized these two species, but this material is in need of revision. UMNH VP26151differs from “*Podocnemis*” *parva* by lacking a preneural, but we have little confidence in the actual presence of this element in “*Podocnemis*” *parva*. UMNH VP26151otherwise differs by possessing a neural VII, a narrower vertebral I that barely laps onto peripheral I, and significantly more enlarged anterior marginals. UMNH VP26151differs from “*Podocnemis*” *judea* by lacking a preneural, by exhibiting a midline contact of costals VII, and by possessing costals VIII, but, once again, we are not confident about the highly unusual morphology reported for “*Podocnemis*” *judea*. UMNH VP26151otherwise has narrower vertebrals, but significantly larger marginals.

Only a single shell, Yale Peabody Museum of Natural History (YPM) 3608, has been reported from the Coniacian or Turonian globally and referred to *Chedighaii barberi* (Gaffney & Zangerl, 1968; Gaffney, Tong & Meylan, 2006). UMNH VP26151differs from YPM 3608 by being much smaller, having more elongate neurals, more extensive axillary buttresses that contact costal II, a broader anterior plastral lobe with more parallel lateral margins, and smaller extragulars.

UMNH VP26151finally differs autapomorphically from all other described bothremydids by having a clear contact of the axillary buttresses with costal II and by having a surface pattern that consists of irregular grooves along the center of the shell that concentrate towards the margins to form irregular polygons. We therefore feel justified to name a new taxon, *Paiutemys tibert* gen. et sp. nov.

### Phylogenetic relationships

We here reorganized the character/taxon matrix of Gaffney, Tong & Meylan (2006) to allow the multistate characters that form a morphocline to be ordered. The fact that our reorganizations does not effect the actual character observations made by Gaffney, Tong & Meylan (2006) is easily confirmed, as the revised matrix produces the same tree when the characters are left unordered.

Preliminary analysis revealed that *Paiutemys tibert* is placed near the base of Bothremydidae near the European taxa *Foxemys mechinorum* and *Polysternon provinciale*. We therefore expanded the taxonomic sample of our analysis to include the Campanian *Elochelys perfecta* and Maastrichtian *E. convenarum*, which previous analyses have shown to be affiliated with Bothremydidae (Gaffney, Tong & Meylan, 2006). We furthermore added the basal bothremydid *Atolchelys lepida* from the Barremian of Brazil (Romano et al., 2014) and *Chupacabrachelys complexus* from the Campanian of the Texas (Lehman & Wick, 2010), because of their potential biogeographic significance, and *Foxemys trabanti* from the Santonian of Hungary (Rabi, Tong & Botfalvai, 2012), because it of its potential phylogenetic significance. We did not activate the Cenomanian “*Podocnemis*” *parva*, as scored by Gaffney, Tong & Meylan (2006), because this taxon is in dire need of revision and because we have little confidence in the highly unusual morphology reported for this taxon.

We ran the expanded character matrix twice, once with ordered characters and once with the characters unordered. The resulting trees are similar in many respects, but differ substantially in others. We briefly highlight aspects we find notable using the new phylogenetic nomenclature we propose herein:

(1) The ordered analysis places *Sankuchemys sethnai* within Kurmademydini, as originally proposed by Gaffney, Tong & Meylan (2006), and confirms the placement of *Atolchelys lepida* at the base of Bothremydidae, as originally suggested by Romano et al. (2014). The unordered analysis places these taxa in an unresolved basal bothremydid polytomy. The global pelomedusoid analysis of Cadena (2015) retrieved *Atolchelys lepida* outside of Bothremydidae.

(2) Both analyses confirm the monophyly of Cearachelyini as originally proposed by Gaffney, Tong & Meylan (2006). This grouping was not retrieved by the analysis of Romano et al. (2014), but was recovered by the global analysis of Cadena (2015).

(3) The unordered analysis assigns all derived bothremydines to Cearachelyini, Bothremydini, and Taphrosphyini in an arrangement similar to those of Gaffney, Tong & Meylan (2006) and Pérez García (2016). The ordered analysis, by contrast, moves a number of taxa from Bothremydini to the lineage leading to the clade formed by Bothremydini and Taphrosphyini. A similar result was also retrieved by the bothremydid subanalysis of Cadena (2015), though with the use of unordered characters.

(4) The unordered analysis retrieves *Araiochelys hirayamai* within Bothremydini, as found by Gaffney, Tong & Meylan (2006), Romano et al. (2014), Cadena (2015), and Pérez García (2016). The ordered analysis retrieves the novel placement of this taxon within Taphrosphyini.

(5) As initially suspected by Lehman & Wick (2010), *Chupacabrachelys complexus* is retrieved within Taphrosphyini in both analyses.

(6) In contrast to Gaffney, Tong & Meylan (2006), *Elochelys convenarum* and *Elochelys perfecta* are also retrieved within Taphrosphyini in both analyses.

(7) In both analyses, *Paiutemys tibert* is retrieved as sister to a European clade consisting of *Polysternon provinciale*, *Foxemys trabanti*, and *Foxemys mechinorum*, but, as described above, this clade is placed within Bothremydini in the unordered analysis, but outside of Bothremydini and Taphrosphyini in the ordered analysis.

Our analysis is similar to previous analyses (e.g., Rabi, Tong & Botfalvai, 2012; Romano et al., 2014; Cadena, 2015; Pérez García, 2016) by being based on the character/taxon matrix of Gaffney, Tong & Meylan (2006). All differences in the topology are therefore based on changes either to the ingroup or the use of ordered characters. In that regard, it is notable that the results of our ordered analysis converge upon those of the unordered analysis of Cadena (2015) by recognizing a “stem lineage” leading to the clade formed by Bothremydini and Taphrosphyini. Although the placement of *Paiutemys tibert* within Bothremydidae depends on the use of ordered characters in our analysis, it is universally retrieved towards the base of the clade, which is broadly consistent with the stratigraphic record, as it is one of the oldest known representatives of Bothremydinae.

### Biogeography

Some subclades of bothremydids were partially adapted to brackish to marine waters and therefore dispersed more easily than most other freshwater groups of pleurodires. Rabi, Tong & Botfalvai (2012) provided an initial paleobiogeographic assessment for *Bothremydini* (our Bothremydini + the *Paiutemys tibert* clade) and concluded that Europe and North America were each colonized two times by African bothremydids.

A more global view of bothremydids confirms the conclusion of Rabi, Tong & Botfalvai (2012) for the addressed subclades, but adds additional data for other portions of the tree. The topologies resulting from the analyses using ordered (Fig. 5) and unordered characters (Fig. 6) reveal that basal bothremydids are known from Africa, South America, and India. At the very least, this confirms that the group originated in Gondwana during the early Cretaceous and only secondarily colonized Europe and North America during the late Cretaceous. A literal interpretation of these trees within a parsimony framework implies that bothremydids migrated from Gondwanan to Europe twice, as already highlighted by Rabi, Tong & Botfalvai (2012), in particular the ancestor of *Rosasia soutoi* at some point prior to the Campanian and the ancestor of the clade giving rise to *Elochelys*, *Foxemys*, and *Polysternon* spp. prior to the Santonian, a conclusion more recently confirmed by Pérez García (2016) as well. Our extended data set implies that at least five separate clades furthermore invaded North America from Gondwana. These migrations include the previously established ancestors of *Chedighaii* prior to the Campanian and *Bothremys cooki* prior to the Maastrichtian, and the newly established ancestors of *Taphrosphys sulcatus* prior to the Paleocene and those of *Paiutemys tibert* prior to the Cenomanian and that of *Chupacabrachelys perplexus* prior to the Campanian. *Paiutemys tibert* has no close relationships with any other North American bothremydids and therefore represents an early, independent dispersal event to North America.

Although these biogeographic conclusions might be refined through the use of various biogeographic models, we here note that the vast majority of bothremydines are found in near-shore marine deposits surrounding the Atlantic Ocean (Gaffney, Tong & Meylan, 2006), in contrast to basal bothremydids, which are still retrieved from continental sediments (Gaffney, Tong & Meylan, 2006; Romano et al., 2014). Just as a literal interpretation of the fossil record of chelonioid turtles may result in a perplexingly complex set of dispersal events across oceanic barriers, a literal interpretation of bothremydines is becoming increasingly complex. We therefore suggest that bothremydines should simply be viewed as circum-Atlantic and that the biogeography of the group should not be over interpreted as consisting of an endless series of dispersal events.

##  Supplemental Information

10.7717/peerj.2502/supp-1 File S1 Modified list of character utilized in the phylogenetic analysis

10.7717/peerj.2502/supp-2 File S2 Character taxon matrix in nexus format

10.7717/peerj.2502/supp-3 File S3 Common synapomorphies mapped onto the phylogenetic hypothesis resulting from the use of ordered characters

10.7717/peerj.2502/supp-4 File S4 Common synapomorphies mapped onto the phylogenetic hypothesis resulting from the use of unordered characters

## Additional Information and Declarations

### Data Availability

The following information was supplied regarding data availability: The raw data has been supplied as Supplemental Files.

### New Species Registration

The following information was supplied regarding the registration of a newly described species: Paiutemys LSID: urn:lsid:zoobank.org:act:03EDB775-F4DA-46CC-9DB7- 4C221B79F211; Paiutemys tibert LSID: urn:lsid:zoobank.org:act:D04DD2F1-97EE-4AE4-A578-3F1180BF7AB9; Publication LSID: urn:lsid:zoobank.org:pub:8322CE62-F815-49A9-AC13- 4169E4CD3F43.
