# Supplementary material for: An early bothremydid (Testudines, Pleurodira) from the Late Cretaceous (Cenomanian) of Utah, North America
Source: PeerJ. 2016 Sep 28;4:e2502. doi: 10.7717/peerj.2502 (PMC5045886; doi:10.7717/peerj.2502)
Supplement: File S1 [file peerj-04-2502-s001.docx]

**Supplemental File S1: Final list of characters used in phylogeny analysis.**

1–13: see characters 1–13 of Gaffney et al. (2006).

14: temporal emargination: 0 = absent to minor; 1 = intermediate; 2 = extreme.

15–27: see characters 15–27 of Gaffney et al. (2006).

28: septum orbitotemporale: 0 = absent; 1 = postorbital wall at least partially open; 2 = postorbital wall present

29–31: see characters 29–31 of Gaffney et al. (2006)

32: dorsal process of premaxillae: 0 = present, meeting nasals; 1 = present, meet prefrontals; 2 = absent.

33–37: see characters 33–37 of Gaffney et al. (2006).

38: maxilla-quadrate contact: 0 = absent, clearly separated; 1 = absent, narrowly separated; 2 = present.

39: cheek emargination: 0 = little or no emargination; 1 = deep emargination present; 2 = extremely deep emargination present, quadratojugal absent.

40: orbital-narial bar width: 0 = extremely narrow; 1 = intermediate, equal to or slightly less than orbit diameter; 2 = broad, less than twice the width of the orbit; 3 = extremely broad, more than twice the width of the orbit.

41–50: see characters 41–50 of Gaffney et al. (2006)

51: antrum postoticum: 0 = absent; 1 = small; 2 = moderate to large.

52–59: see characters 52–59 of Gaffney et al. (2006)

60: relative placement of condylus mandibularis: 0 = anterior to basioccipital-basisphenoid suture; 1 = posterior to basioccipital/basisphenoid suture; 2 = posterior to condylus occipitalis.

61–84: see characters 61–84 of Gaffney et al. (2006)

85: exoccipital-quadrate contact: 0 = absent; 1 = narrow; 2 = extensive.

86–103: see characters 86–103 of Gaffney et al. (2006)

104: basisphenoid–quadrate contact: 0 = absent; 1 = present and narrow; 2 = present and wide.

105–138: see characters 105–138 of Gaffney et al. (2006)

139: nuchal bone width: 0 = width twice the length, or greater; 1 = width greater than length, but less than twice; 2 = width equals length; 3 = width less than length.

140–143: see characters 140–143 of Gaffney et al. (2006)

144: presence of four sided neural: 0 = present; 1 = absent.

145: position of four sided neural: 0 = neural I; 1 = neural II; 2 = neural III.

146–147: see characters 145–146 of Gaffney et al. (2006)

148: peripheral I – costal I contact: 0 = absent; 1 = present, narrow, contact half the anterior margin of peripheral I; 2 = present, broad, contact less than half the anterior margin of peripheral I.

149: costal contact of axillary process: 0 = absent; 1 = present, but far from costal II; 2 = present, but approximating the margin of costal II; 3 = present and crossing over to costal II.

150–176: see characters 149–175 of Gaffney et al. (2006).
