# Supplementary figures and images for: An early bothremydid (Testudines, Pleurodira) from the Late Cretaceous (Cenomanian) of Utah, North America
Source: PeerJ. 2016 Sep 28;4:e2502. doi: 10.7717/peerj.2502 (PMC5045886; doi:10.7717/peerj.2502)

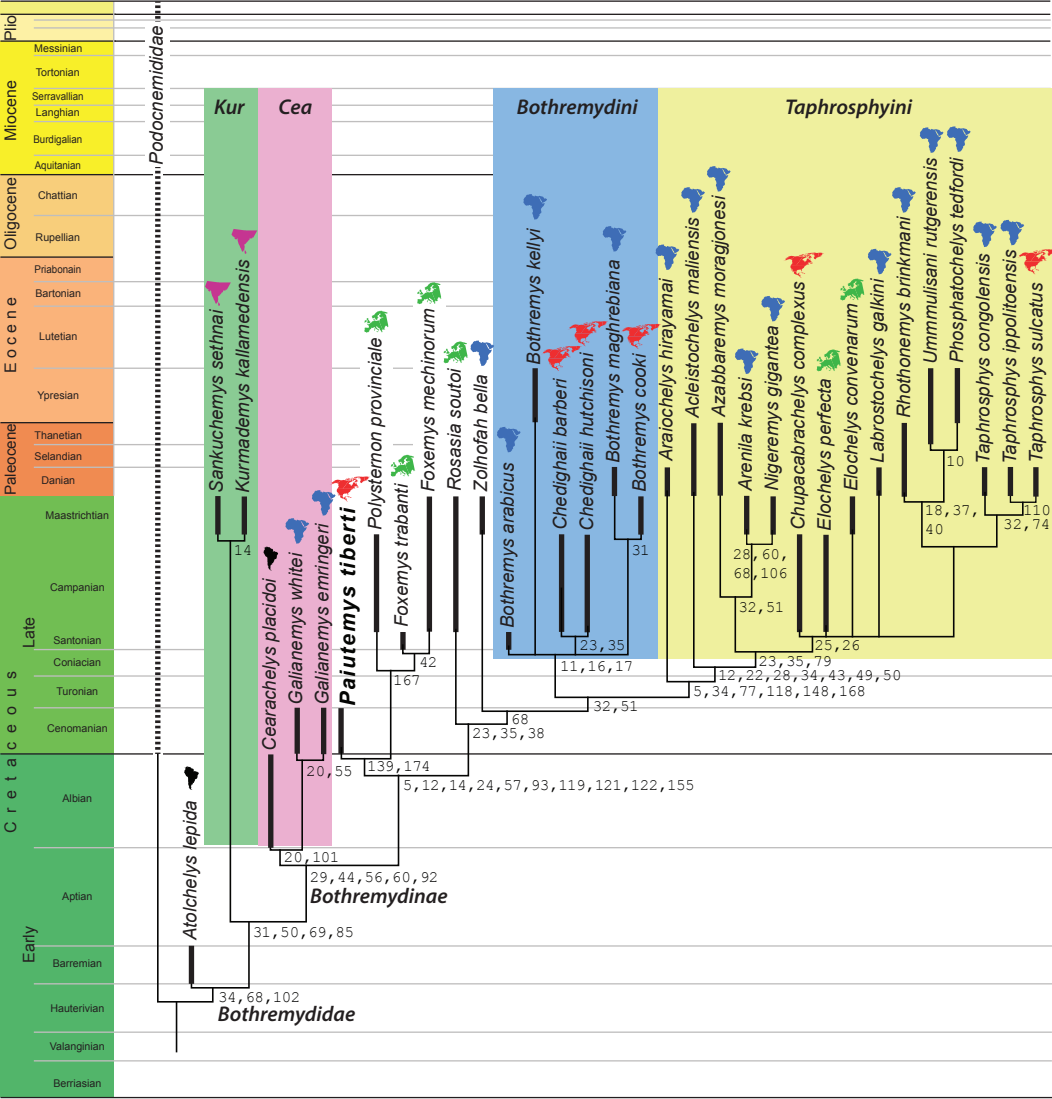

Supplement: File S3 [file peerj-04-2502-s003.pdf]

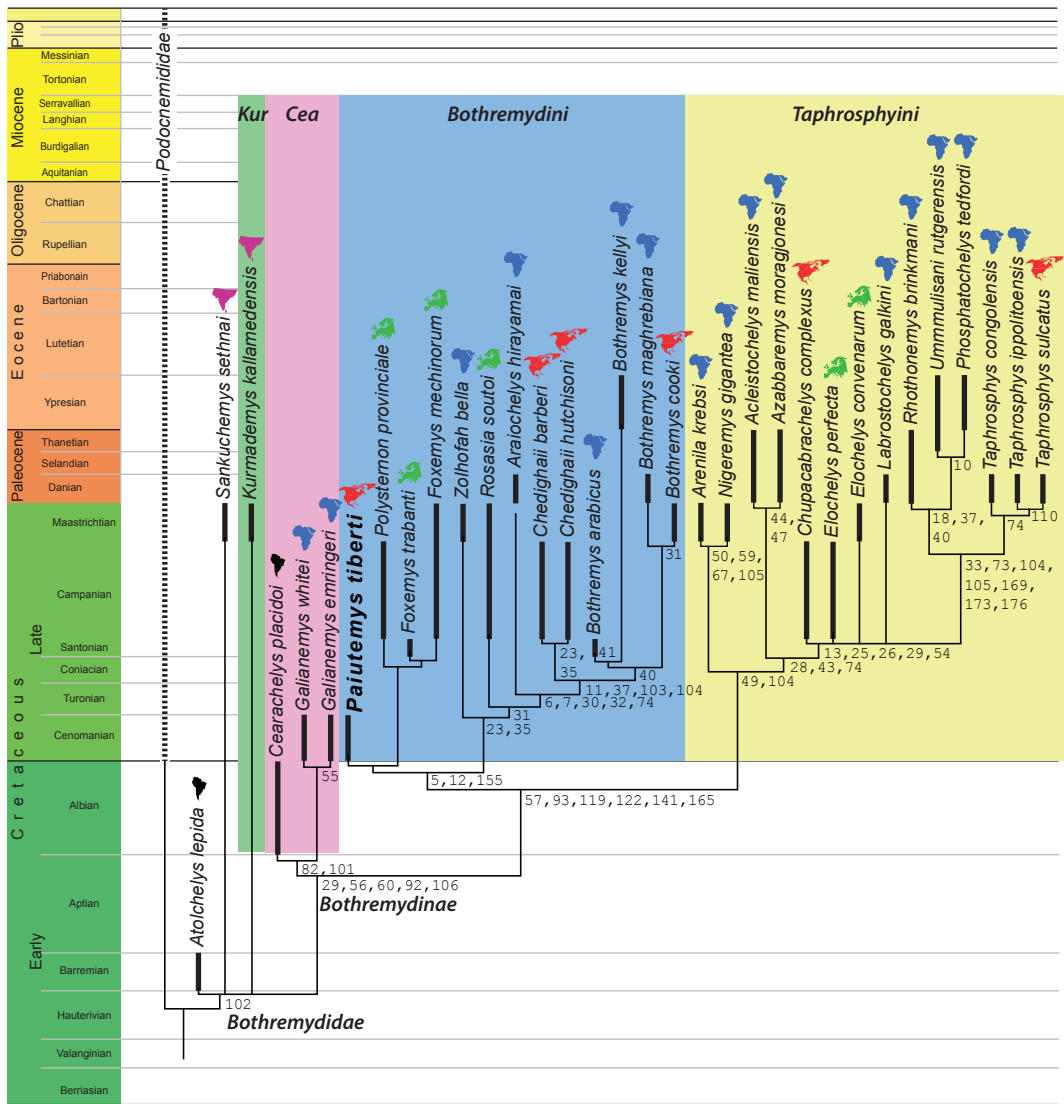

Supplement: File S4 [file peerj-04-2502-s004.pdf]
